# *Cucurbita maxima* Seeds Reduce Anxiety and Depression and Improve Memory

**DOI:** 10.1155/2023/7509937

**Published:** 2023-03-22

**Authors:** Shahana Wahid, Ali Alqahtani, Rafeeq Alam Khan

**Affiliations:** ^1^Department of Pharmacology, University of Karachi, Karachi, Pakistan; ^2^Faculty of Pharmacy, Benazir Bhutto Shaheed University Lyari, Karachi, Pakistan; ^3^Department of Pharmacology, College of Pharmacy, King Khalid University, Guraiger, Abha 62529, Saudi Arabia; ^4^Faculty of Pharmacy, Ziauddin University Education City, Link Road, Karachi, Pakistan

## Abstract

The current study was planned to assess the neuropharmacological benefits of the *Cucurbita maxima* seed. These seeds have been conventionally used for the nutritional as well as amelioration of various diseases. However, there was a need to provide a pharmacological basis for such use. Four central nervous system-related functions, that is, anxiety, depression, memory, and motor coordination, were evaluated, and the levels of brain biogenic amines were also assessed. Anxiety was evaluated through selected experimental models, such as light and dark apparatus, elevated plus maze, head dip, and open field test. The head dip test was mainly used to assess exploratory behavior. Depression was assessed by two animal models, that is, the forced swim test and tail suspension test. Memory and learning ability were assessed by the passive avoidance test, stationary rod apparatus, and Morris's water maze test. Motor skilled learning was assessed by stationary rod and rotarod apparatus. Reversed phase high-pressure liquid chromatography was used to determine biogenic amine levels. Results reveal that *C. maxima* exhibited anxiolytic and antidepressant effects with memory improvement. There was a reduction in the weight of the animal following chronic administration. Furthermore, no remarkable effects were observed on motor coordination. Norepinephrine was found elevated, which may be linked to its antidepressant effects. These biological effects of *C. maxima* may be due to the presence of secondary metabolites, such as cucurbitacin, beta-sitosterol, polyphenolic compounds, citrulline, kaempferol, arginine, *β*-carotene, quercetin, and other antioxidants. The outcomes of the present study authenticate that the chronic use of *C. maxima* seeds reduces the intensity of neurological problems like anxiety and depression.

## 1. Introduction

Various neurological conditions, such as multiple sclerosis, traumatic brain injuries, and dementia, might be one of the causes of depression and anxiety. Dementia is a condition that affects the person's ability to execute routine life activities interfering with decisions of persons. To rule out the degree of dementia, the familiar paradigm is to evaluate one's ability to new learning and memory by certain models. Learning is an initial course of forming novel memories in the brain from an initial experience of previously learned events. It involves encrypting, storing, retrieving, and retaining the event in memory or forgetting [1]. Short-term memory is the storage of information for a short period without any repetition, whereas long-term memory is the preservation of data for an extended period due to recurrence. The usual learning procedure is based on the availability of neurotransmitters, such as dopamine, acetylcholine, and 5-hydroxytryptamine (5HT), which stimulate the hippocampus, amygdala, and other brain areas of the cerebral cortex like the sensory, visual, and auditory cortices [2]. Furthermore, several anxiolytics and antidepressant drugs alter learning memory or amnesia.

Cholinesterase inhibitors and dopamine agonists are considered the first line of treatment for the management of dementia [3]. Furthermore, some neurodegenerative disorders, such as Alzheimer's disease (AD), have progressive dementia either due to cholinergic neuronal loss or decreased levels of acetylcholine. The first management strategy for AD is to use acetylcholinesterase (AChE) inhibitors, which largely yield an effect by enhancing acetylcholine levels in the brain. Herbal remedies are traditionally popular for the treatment of various ailments because of the bioactive compounds in them.

Anxiety is a repeated episode of intense uncontrolled feelings of fear, confusion, tachycardia, and various panic symptoms. The short period of the depressive phase reaction is a normal reaction to everyday stress. However, if episodic symptoms of uneasiness continue that hinder the person's daily activity, then it is considered an anxiety disorder [4]. The major type of anxiety is generalized anxiety disorder. Furthermore, it is also associated with various depressive or behavioral disorders making management difficult [5]. There is a need to discover drug molecules having selectivity for 5-HT1A receptor antagonists and benzodiazepine type suppressant characteristics [6].

Depression is another psychological issue having persistent opposite depressive symptoms for more than 2 weeks. Depression is manifested by a feeling of sadness, guilt, tiredness, diminish concentration, loss of interest, low confidence, and disturbance in appetite or sleep. According to the Diagnostic and Statistical Manual, there are various types of depression, such as persistent depressive disorder, major depression, situational or seasonal affective disorder, postpartum depression, bipolar depression, psychotic depression, premenstrual dysphoric depression, and atypical depression [7].

Depression is a complex disorder that refers to the experience of feeling sadness and some heterogeneous associated symptoms that vary broadly characterized by loss of positive thoughts. Behavioral attributes of depression must incorporate the controlling variables at both interoceptive and environmental levels. These effects themselves are not problematic but rather adaptive, but if it becomes chronic, dysregulated, or maladaptive through several etiologies, then it needs to be treated. Moreover, depressed people commonly exhibit depressive-related behaviours including overthinking and social avoidance. This event may be negatively strengthened and create a cycle of increasing negative thoughts and avoidance, maliciously leading to depression [8]. According to the United States National Research Council and Institute of Medicine, various biological and environmental factors pose the person to depression including low age, genetic tendency, neurological or neuro-endocrinological, hormonal, and immunological. All these factors are cumulatively triggered by environmental factors such as stressful experiences of vulnerable persons due to psychosocial and biological circumstances. The presence of other medical and psychological disorders also worsens depression making it difficult to treat [9]. The initial antidepressant drugs were initially discovered based on hypotheses related to central nervous system (CNS) impairment of norepinephrine levels in the 1950s. After the revolution in drug discovery in neuroscience and genetics, new antidepressant drugs have been discovered, which cleared the basic mechanisms of depression and mechanism of the drug showing that the monoaminergic system is one of the bases of these mechanisms along with multiple interactions with other brain regulatory systems [10].

*Cucurbita maxima* was selected to confirm its memory-enhancing effect as several neuroprotective and antioxidant substances have been reported in the literature. *C. maxima* is a plant of Cucurbitaceae important for growth and immunity [11]. Besides its nutritional value, it is also a good source of important metabolites, such as phenolic glycosides [12], carotenoids, *γ*-aminobutyric acid [13, 14], flavonoids, alkaloids, tannins, saponins, and terpenoids [15]. It has been reported that it has hypocholesteremic, anthelmintic, hypotensive, hypoglycaemics, and antiperoxidative properties [16, 17]. Its use can also relieve symptoms of benign prostatic hyperplasia and some anxiety disorders [18]. Pectin isolated from *C. maxima* has the highest cytoprotective and antioxidant effect against reactive oxygen species generation in different cell lines [19, 20]. *C. maxima* seeds are supposed to have a high content of magnesium, which may behave as a N-methyl-D-aspartate (NMDA) receptor blocker, a very good pain killer specifically effective in nerve pain and have a euglycemic effect on diabetes decreasing type 2 diabetes generation in a healthy individual. Furthermore, it is also reported that a daily intake of 400 mg of magnesium oxide acts as a muscle relaxant. Therefore, it may relieve anxiety by relaxing the patients. Thus, due to these reported effects, *C. maxima* seeds were selected to evaluate their effects on the reduction of neurodegenerative disorders.

## 2. Materials and Methods

### 2.1. Collection of Seeds and Extraction

The seeds of *C. maxima* were bought from the local market and identified by the Plant Conservation center at the University of Karachi, Karachi, Pakistan, as GH #9501. The research was performed after approval of the advanced studies board, University of Karachi. The seeds of *C. maxima* were soaked in the proportion of 1 kg in 1.5 L using ethanol for 21 days. Seeds were kept in airtight ambered bottles during maceration followed by filtration and evaporation of the solvent. The extract was stored at 4°C till use after freeze-drying.

### 2.2. Experimental Design

The study was performed on healthy mice of either sex bred in the animal house, Department of Pharmacology, the University of Karachi, after acclimatization for 1 week. Mice were distributed into six groups having 10 mice per group kept in polycarbonate cages at 25 ± 2°C with access to food and water ad libitum. Animal of the control group received 5% Dimethyl sulfoxide (DMSO), two groups were given standard drugs diazepam 3 mg/kg [21] and imipramine 30 mg/kg [22], whereas mice of the test groups received ethanol extracts of *C. maxima* at doses of 50, 100, and 200 mg/kg for 30 days. The oral route of administration was selected to give DMSO, standard drugs, and seed extracts daily between the fixed time of 12 am to 1 pm. Behavioral experimentations were primarily conducted on days 1, 8, and 21 using different models for anxiety, depression, and memory. The behavioral sessions were recorded using an android mobile camera. At the end of the study, five mice from each group were sacrificed to determine the level of biogenic amines.

### 2.3. Chemicals

Chemicals purchased during the study were DMSO, imipramine hydrochloric acid (HCl; Sigma‑Aldrich, Headquarter Munich, Germany), Diazepam (Roche, Pakistan), some high-pressure liquid chromatography (HPLC) standards, such as homovanillic acid (HVA), noradrenaline (NA), adrenaline hydrochloride, 5HT, 5-hydroxy indole acetic acid, ethylene-diamine-tetra-acetic acid disodium salt, 3,4 dihydroxy-phenyl acetic acid (DOPAC), dopamine hydrochloride (DA), and HCl. Furthermore, some more chemicals were HPLC grade chemicals, such as methanol, acetonitrile, and deionized water. For disinfecting the apparatus after each reading, ethanol was used.

### 2.4. Model for Anxiety

#### 2.4.1. Light and Dark Test

This model was employed to determine the anxiolytic ability of seed extracts by determining the preference of animals to be in the lighter compartment. This model has two equal compartments of 20 cm × 20 cm. The lighter compartment was brightened with a 100 V bulb, whereas the dark compartment had dark glasses that led to a darker region. The mice were introduced to a darker zone, and 5 minutes test sessions were performed during mice were freely moved between two compartments through an intermediate small gate. The anxiolytic activity was determined after measuring the time spent by mice in the light compartment, their percentages, and number of transitions. An increment in the percentage of time spent in the light area was suggestive of anxiolytic activity [23].

#### 2.4.2. Elevated Plus Maze (EPM)

Subsequently, mice were employed in EPM on the eighth and twenty-first days of dosing. The EPM apparatus comprises two open arms (50 cm × 10 cm) and two closed arms (50 cm × 10 cm × 38 cm) linked to the central area (10 cm × 10 cm) at a height above 50 cm from the ground. Mice were placed in the central area towards the open arm and behavior was recorded for a 5-minute test session. Behavior measured were several entries into the open arm as well as the closed arm and time spent by the animal in the open arm and closed arm. An increment in time spent in the open area and the number of entries in the open arm was considered an anxiolytic activity. The anxiety index was calculated using the following formula. Anxiety in mice would be present if the result of the anxiety index was found from 0.6 to 1.0, whereas a reduction in the index exhibited an anxiolytic effect [24].

(1) $$\begin{array}{c} \text{Anxiety index}=1-\left(\text{time in open arm}/\text{total time}\right)+\left(\text{entries of open arm}/\text{total entries}\right). \end{array}$$

#### 2.4.3. Head Dip Test

This test includes a white square wooden box of 35 cm × 45 cm × 45 cm containing equally spaced and sized three holes located at each side. Mice were introduced for 5 minutes session and several head dips through such holes were noted on the 8th and 21 days [25]. The increment in head dipping in this model reveals a rise in anxiety, whereas a decline in head dipping shows the anxiolytic effect of extracts [26].

#### 2.4.4. Open Field Test Apparatus

It is a gold standard apparatus assessing different behaviors of rodents, such as anxiety, memory, exploration, and even depression. It consists of a plexiglass cube of 75 cm × 75 cm × 40 cm with marked flooring divided into 25 boxes, each of 15 cm × 15 cm with a central area demarcation of 30 cm × 30 cm. Mice were introduced in the center to explore the open field test (OFT) apparatus for 10 minutes session [27, 28]. During the test session, the parameters measured were the total distance covered by mice, the number of entries in the center, and their duration, rearing frequency, and duration. A reduction in the total distance shows a reduction in motor activity that may be due to sedative effects. An increment in time spent in center or center entries was revealing the anxiolytic activity. An increment in the frequency or duration of rearing shows high expressive and thoughtful behavior and an anxiety reduction. Recently, researchers related increment in rearing behavior with antidepressant effects [29].

### 2.5. Models for Memory

#### 2.5.1. Passive Avoidance Test

Passive avoidance test (PAT) test is used for rapid assessment of the fear disinclination memory of mice. The escaping phenomenon was assessed using similar light and black apparatus having a grid floor and guillotine gate. The dark section's grid floor is attached to an electric source for giving tolerable foot electric shocks. The test was accomplished in three phases, i.e., habituation, education, and test periods. The mice were introduced in the illuminated section for 300 seconds on the first and second days of habituation facing the dark area with the guillotine door open. When the mice entered the dark booth, the door was shut down, and a foot jolt of 0.6 mA for 0.5 seconds was given through the grid floor [31]. During the test session, the mice were re-introduced into the apparatus after three hours, twenty-four hours, the eighth day, and the twenty-first day to assess short-term and long-term memories. Mice were placed in the illuminated section keeping the gate open to provide free access to the dark disinclination section. The latency to re-enter the black section was observed escaping electric shock with a maximum cut-off time of 300 seconds.

#### 2.5.2. Morris Water Maze Test

This test was designed to evaluate the hippocampus-dependent acquisition of short- and long-term spatial learning and memory. This maize comprises rectangular water pool of dimension 60 cm × 30 cm. The test used water immersed fixed central stage of dimension 15 cm × 13 cm hidden by starchy at 25°C. In the education session, the stage was kept visible by maintaining the water below the stage. Mice were introduced in the pool and allowed to locate the stage. Four training sessions were performed for four consecutive days so that mice learned to discover the stage within a short period. The mice were tested on the first day, the eighth day, and the twenty-first day. A reduction in time was considered an improvement in special learning [33].

### 2.6. Motor Skilled Learning and Coordination

#### 2.6.1. Effects on Motor Coordination through Rotarod

At present, anxiolytic drugs have effects on grip strength and muscle; therefore, in the current study, grip strength and muscular activity were examined via rotarod apparatus. The rotarod apparatus comprises an enclosed plastic rod of 8 cm × 3 cm. First, mice were given the training to walk on the stationary rods through four successive trails. The test session was performed at two speeds, i.e., 10 and 30 rpm on days 8th and 21st of administration of ethanol extracts. Latency time to keep on the movable rod until mice fall was noted with the cut-off time 180 seconds. An increment in time to keep on the rotating rod was considered an improvement in muscular activity. Drugs recognized to change neuromuscular coordination, such as diazepam, decrease the latency time of mice to keep on the rotating rod [30].

#### 2.6.2. Stationary Rod Test

The education capability was assessed by stationary rod test (SRT) comprised raised steel rods with netted base. Mice were first taught by making them walk on the elevated rod with a networking stage. Animals were trained daily through four training sessions for four consecutive days with each trial of 120 seconds. After completing training, DMSO, standard drugs, and *C. maxima* seed extracts were administered orally for 30 days. The time required by animals to reach the stage was observed on the day first for short-term motor skilled learning, whereas on the eighth day and the twenty-first day for long-term motor skilled learning [32].

### 2.7. Models for Depression

#### 2.7.1. Forced Swim Test

The test was employed to evaluate the anti-depressant effects of the extracts of the seeds on the 22nd day. The apparatus consists of rectangular plexiglass container of dimension 46 cm × 20 cm. Depression was induced in mice through the initial 2 minutes session by the learned helplessness phenomenon, whereas the last 4 minutes of mice's immobility time was noted as a sign of antidepressant activity [34]. Immobility is considered by the absence of the movement of mice except for those that required mice to keep their head out of water. Percent reduction in immobility time was used to assess the antidepressant effect of extracts [35, 36].

#### 2.7.2. Tail Suspension Test

To verify the antidepressant effect, tail suspension method was used on the 22nd day. Animals were suspended with tape with their tails upside down for 6 minutes [37] inducing depression in the initial 2 minutes by the learned helplessness phenomenon. Immobility time was noted in the last 4 minutes, which was associated with antidepressant activity. The immobility time was measured as the absence of the movement of mice to upright themselves. Percent reduction in immobility was considered a degree of antidepressant effect of the extracts [35, 36]. Following the behavioral test, the mice were subsequently sacrificed via cervical dislocation. Their brains were taken out, stored at or below 80°C, and then processed for neurochemical analysis.

#### 2.7.3. Neurochemical Analysis

Biogenic amines were determined by the reversed phase high-performance liquid chromatography or HPLC electrochemical detection (HPLC-EC) method using octadecylsilyl C18 column and methanol as a mobile phase [38]. These stored brains were initially defrosted, homogenized, and followed by extraction of biogenic amines using an extraction medium comprises of perchloric acid (70%). Consequently, two times centrifugations were performed that separated the homogenate. Homogenate was further separated by a reversed-phase column at a constant flow rate (1 mL/minutes) with the help of an HPLC pump. Electrochemical detection of separated biogenic amines was done using their correspondence standards that run simultaneously along with samples (Shimadzu, Kyoto, Japan) at 0.8 V of operating potential.

### 2.8. Statistical Analysis

All result values were calculated as Mean ± SEM by the SPSS statistical software package 26. One way analysis of variance was used followed by a post hoc Dunnet test at *P* < 0.5  and *P* < 0.1.

## 3. Results

### 3.1. Effect on Weight

Table 1 shows the effect of *C. maxima* on weight variation in mice. *C. maxima* seed extracts at 100 and 200 mg/kg exhibited an extremely substantial reduction in weight as compared with their initial weights on the 30th day with a percent reduction in weight by 11% at both doses.

### 3.2. Effect on Memory and Learning

#### 3.2.1. Passive Avoidance Test

**Figure 1:**
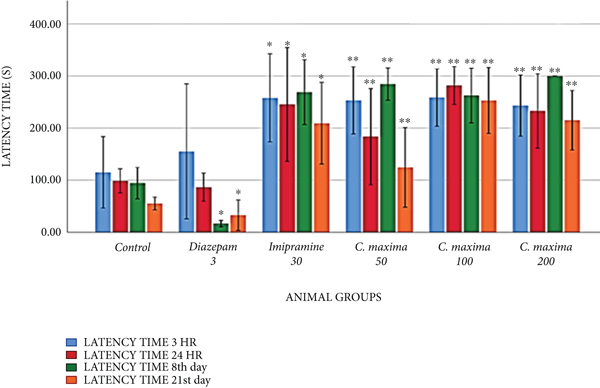
Effect of *C. maxima* on memory (passive avoidance test).

Table 2 and Figure 1 reveal the effects of *C. maxima* seed extract on memory and learning by PAT. *C. maxima* 50 mg/kg exhibited a greatly substantial increment in latency time at 3 hours and 8th day. *C. maxima* 100 mg/kg revealed a greatly substantial increment in reaction time at 3 hours, 24 hours, 8th day, and 21st day. *C. maxima* 200 mg/kg revealed a substantial increment in reaction time at 3 hours and 21st day, whereas an extremely substantial increment in reaction time at 24 hours and 8th day.

Diazepam 3 mg/kg exhibited a significant reduction in latency time on the 8th and 21st days, whereas imipramine 30 mg/kg exhibited a significant increment in latency time at 3 hours, 24 hours, 8th day, and 21st day.

#### 3.2.2. Water Maze Test

**Figure 2:**
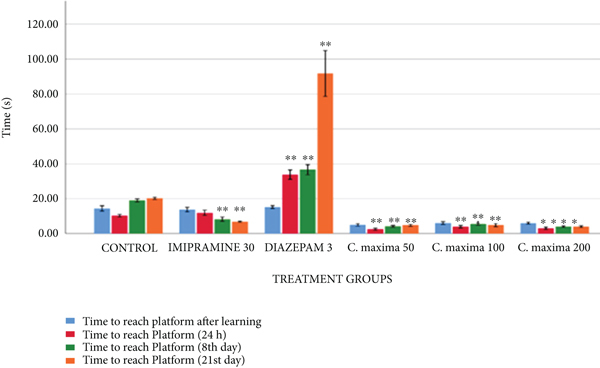
The outcome of *C. maxima* seeds on the memory (water maze test).

Table 3 and Figure 2 reveal the effects of *C. maxima* seed extract on memory and learning by water maze test. *C. maxima* 50 and 100 mg/kg revealed a greatly substantial reduction in time to arrive at the central hidden stage on the eighth day and a substantial reduction in time to arrive at the central stage on the twenty-first day as compared with control. *C. maxima* 200 mg/kg showed a greatly substantial reduction in time to arrive at the stage at twenty-four hours and the eighth day, whereas the substantial reduction in time to arrive at the stage on the twenty-first day. Diazepam 3 mg/kg group revealed a greatly substantial increment in time to arrive at the central hidden stage on the eighth and twenty-first days, whereas imipramine 30 mg/kg revealed a greatly substantial reduction in time to arrive at the central hidden stage on the 8th and 21st days in comparison with control.

### 3.3. Effects on Motor Coordination and Motor Skilled Learning

#### 3.3.1. Rotarod Test

*C. maxima* 100 and 200 mg/kg showed a substantial increment in fall time on the twenty-first day at low speed in comparison with control (Table 4). These *C. maxima* groups showed a greatly substantial increment in fall time on the 8th and 21st days at high speed in comparison with control. Diazepam 3 mg/kg exhibited a greatly substantial reduction in fall time on the 8th day at both rpm, whereas a highly significant reduction in fall on the 21st day at both rpm. Imipramine 20 mg/kg showed a greatly substantial increment in fall time on the 8th and 21st days at high speed in comparison with control.

### 3.4. Stationary Rod Test

**Figure 3:**
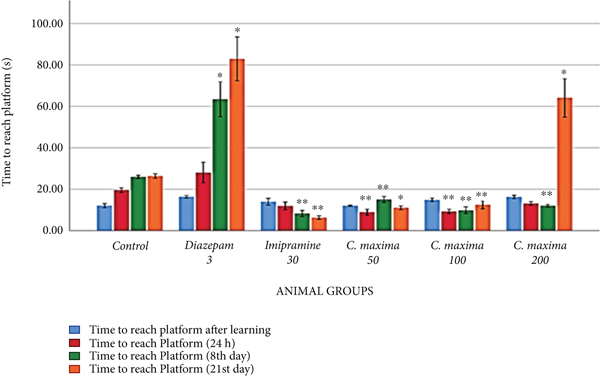
The outcome of *C. maxima* seeds on motor skilled learning and memory (stationary rod).

Table 5 and Figure 3 reveal the effects of *C. maxima* seed extract on memory and learning by SRT. *C. maxima* 50 mg/kg showed an extremely substantial reduction in time to reach the elevated stage at twenty-four hours and the eighth day, whereas a substantial reduction in time to reach the elevated stage on the 21st day in comparison with control. *C. maxima* 100 mg/kg showed a greatly substantial reduction in time to attain the elevated stage on the twenty-four hours, 8th day, and 21st day. *C. maxima* 200 mg/kg revealed a greatly substantial reduction in time to reach the stage on the eighth day and a substantial increment in time to reach the stage on the twenty-first day in comparison with the control. Animals who received diazepam 3 mg/kg exhibited a substantial increment in time to reach the elevated stage on the eighth and twenty-first days, whereas imipramine 30 mg/kg exhibited a substantial reduction in time to reach the elevated stage on the eighth and twenty-first days in comparison with control.

### 3.5. Effect on Anxiety

#### 3.5.1. Light and Dark Model

*C. maxima* 50 mg/kg showed a greatly substantial increment in transitions on the 8th and 22nd day, whereas a greatly substantial increment in the percentage of time spent in the light area on the 21st day in comparison with control.

*C. maxima* 100 200 mg/kg showed an extremely substantial increment in the percentage of time spent in the light area on the 8th and 21st days, whereas *C. maxima* 100 mg/kg showed a greatly substantial increment in transitions on the 8th day in comparison with the control. Animals who received diazepam 3 mg/kg showed a greatly substantial increment in the percentage of time spent in light area and transitions on the 8th and 21st days in comparison with control. Animals given imipramine 30 mg/kg showed a substantial increment in transitions on the 8th day.  Table 6 showed the effects of *C. maxima* seed extracts on anxiety by light and dark models.

#### 3.5.2. Elevated plus Maze

**Figure 4:**
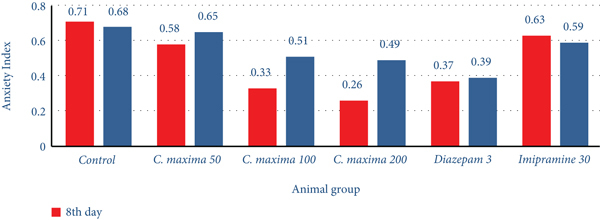
The outcome of *C. maxima* seeds on anxiety index (EPM).

Table 7 and Figure 4 show the effects of *C. maxima* seed extracts on anxiety by eEPM. *C. maxima* 100 and 200 mg/kg showed an extremely substantial and substantial increment in the percentage of time spent in an open area on the 8th and 22nd days, whereas exhibiting a significant increment in transitions on the 8th day as compared with control. These two groups also showed a greatly substantial and substantial reduction in time spent and transitions in closed arms on the eighth and twenty-first days, respectively. *C. maxima* 100 and 200 mg/kg also exhibited a reduction in anxiety index on the eighth and twenty-first days, respectively.

Animals who received diazepam 3 mg/kg exhibited a greatly significant increment in time spent in the open arm on the eighth and twenty-first days as compared with control, whereas exhibiting a significant increment in transitions on the 21st day. Imipramine 30 mg/kg exhibited an increment in time spent in the open arm as compared with the control. The seed extract of *C. maxima* 100 and 200 mg/kg on the 8th day exhibited a significant reduction in the anxiety index as per standard.  Table 6 showed the effects of *C. maxima* seed extracts on anxiety by EPM.

#### 3.5.3. Head Dip Test

*C. maxima* 100 mg/kg showed a greatly substantial reduction in the number of head dips on the 21st day. *C. maxima* 200 mg/kg showed an extremely substantial reduction in the number of head dip on the eighth and twenty-first days in comparison with control animals.

Animals given the diazepam 3 mg/kg exhibited a greatly substantial reduction in the number of head dip on the eighth day while displaying a greatly substantial reduction in the number of head dips on the twenty-first day in comparison with the control. Animals that received imipramine 30 mg/kg revealed a greatly substantial reduction in head dip in comparison with the control group.  Table 8 reveals the effect of *C. maxima* seed extract on several head dips.

#### 3.5.4. Open Field Test

**Figure 5:**
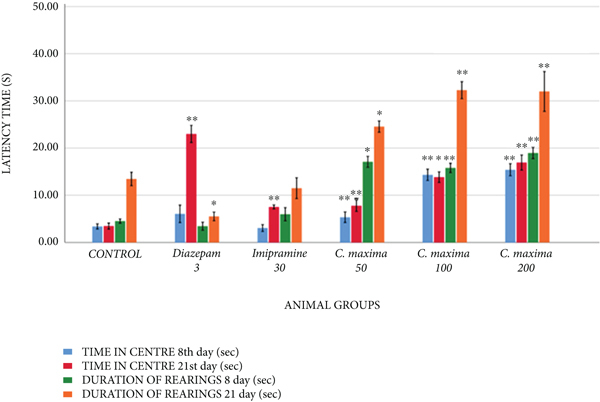
The outcome of *C. maxima* seeds in OFT.

Table 9 and Figure 5 reveal the effects of *C. maxima* seed extract on anxiety by OFT. *C. maxima* 50 mg/kg showed a greatly substantial rise in the total distance on the eighth day; significant and greatly substantial rise in center entries on the 8th and 21st days, respectively; significant and greatly significant increment in the duration of rearing's at the 8th and 21st days in comparison with control, respectively (Table 8). *C. maxima* 100 and 200 mg/kg showed an extremely substantial increment in the total distance on the 8th day; greatly significant increment in center entries, center time; number, and duration of rearing 8th and 21st days in comparison with control. Diazepam 3 mg/kg exhibited a significant reduction in the total distance on the 21st day; a greatly significant increment in center entries along with center time on the 21st day; a significant reduction in the number and duration of rearing's on the 21st day as compared with control. Imipramine 30 mg/kg exhibited an extremely significant reduction in center entries on the 21st day and an extremely significant increment in numbers of rearing's at the 8th and 21st days in comparison with the control.

#### 3.5.5. Effect on Depression

When evaluating the antidepressive research, two models were used. During this examination, every mouse survived. None of the mice drowned when tested using the forced swim test or the tail flick method.

#### 3.5.6. Forced Swim Test

*C. maxima* 50, 100, and 200 mg/kg showed an extremely substantial reduction in immobility time with a percent reduction of 36%, 35.4%, and 49.3%, respectively, on the 22nd day in comparison with the control. Imipramine 30 mg/kg shows maximum antidepressant activity, that us, 54.1% as compared with control in FST.

#### 3.5.7. Tail Suspension Test

**Figure 6:**
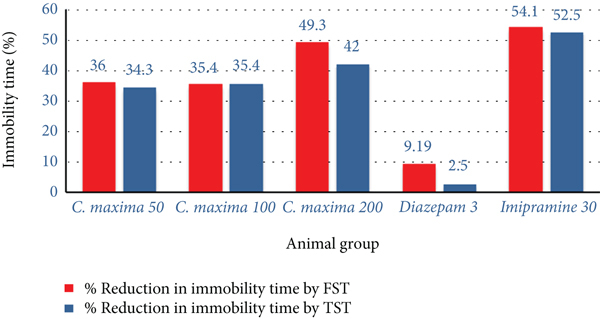
Comparison of reduction in immobility by *C. maxima* seed extract (FST and TST).

*C. maxima* 50, 100, and 200 mg/kg showed an extremely substantial reduction in immobility time with a percent reduction of 34.3%, 35.4%, and 42%, respectively, on the 22nd day in comparison with control. While imipramine 30 mg/kg showed an extremely substantial reduction in immobility time with a percent reduction of 52.5% on the 22nd day as compared with the control (Table 10 and Figure 6).

#### 3.5.8. Brain Biogenic Amines Evaluation

**Figure 7:**
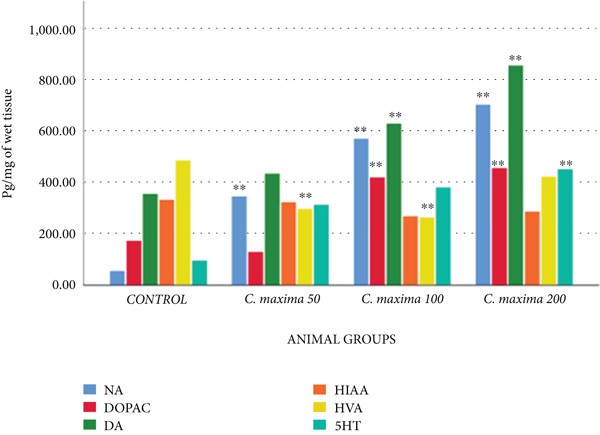
Levels of biogenic amines in the brain.

Table 11 and Figure 7 show the effects of *C. maxima* seeds on brain biogenic amines at selected doses. *C. maxima* 50 mg/kg revealed an extremely substantial increment in concentration of NA and an extremely significant reduction in the concentration of HVA in the brain on the 30th day in comparison with control. *C. maxima* 100 mg/kg revealed an extremely substantial increment in concentration of NA, DOPAC, and DA, whereas an extremely significant reduction in the concentration of HVA in the brain on the 30th day in comparison with the control. *C. maxima* 200 mg/kg revealed a greatly substantial increment in concentration of NA, DOPAC, DA, and 5HT in the brain on the 30th day in comparison with the control.

## 4. Discussion

Herbal medicines are playing a crucial role in the prevention and treatment of various disorders as these are the main sources of secondary metabolites. The current study was designed to evaluate the pharmacological activity of *C. maxima* seeds extract to rule out their role in weight reduction, anxiety, depression, and memory amelioration. Diazepam 3 mg/kg exhibited weight gain, whereas weight gained was more pronounced in the case of imipramine 30 mg/kg (16%). A reduction in weight by more than 10% from initial body weight is a remarkable change in weight which was observed by *C. maxima* at 100 and 200 mg/kg. The weight reduction by *C. maxima* may be due to the presence of high content of steroidal anti-inflammatory substances like cucurbitacin A, B, C, and D and the absence of cholesterol and saturated fatty acids. Furthermore, *C. maxima* seed extracts also revealed a marked reduction in spontaneous activity, pain response, touch response, corneal and light responses, grip strength, at 50, 100, and 200 mg/kg doses, whereas there was marked increment in the balance beam light in the light and dark model. *C. maxima* at 100 mg/kg showed maximum anxiolytic activity, that is, 58.9% and 55% on the 8th and 21st days, respectively, equivalent to diazepam (54%). In the EPM model, the maximum reduction in anxiety index was observed at 200 mg/kg dose on the 8th day, that is, 0.26, whereas diazepam shows a significant reduction in anxiety index, that is, 0.37 and 0.39 at the 8th and the 21st day. Imipramine exhibited no remarkable reduction in anxiety in any model of anxiety.

In the OFT model, the effects of *C. maxima* were almost comparable with the control in terms of total distance covered. Diazepam exhibited a significant reduction in total distance covered by animals on the 21st day, whereas *C maxima* extract and imipramine have no effect on the reduction of total distance covered by the animal on both the 8th and 21st days. Despite the reduction of total distance, diazepam significantly increased the center time and center entries verifying its anxiolytic effect. Imipramine only increases center entries as well as rearing showing improved learning and exploration. However, *C. maxima* exhibited more rearing duration as compared with the imipramine 3 mg/kg. An increase in rearing behaviors shows improved learning, whereas an increase in center activity shows an anxiolytic effect.

In the PAT, *C. maxima* at 50 mg/kg displayed an extremely substantial rise in latency time to enter the punished area at 3 hours and 8th day. *C. maxima* at 100 mg/kg displayed an extremely substantial rise in reaction time at 3 hours, 24 hours, 8^th^ day, and 21st day. *C. maxima* at 200 mg/kg revealed a substantial rise in reaction time at 3 hours and 21st day, whereas a greatly substantial increase in reaction time at 24 hours and 8th day. Diazepam exhibited a significant reduction in latency time to enter a punished dark box due to its amnesic effect. *C. maxima* extracts revealed no such effects. Imipramine 30 mg/kg showed a significant increment in latency time at 8 hours, 24 hours, 8th day and 21st day.

In the water maze test, *C. maxima* 50 and 100 mg/kg showed an extremely substantial fall in time to arrive at the stage on the eighth day and a substantial fall in time to attain the stage on the twenty-first day in comparison with the control. *C. maxima* 200 mg/kg showed an extremely significant decrease in time to reach the stage at a time at 24 hours, and 8th day, whereas a significant decrease in time to reach the stage on the 21st day in comparison with control that is comparable with imipramine. Diazepam significantly increases the time to reach the platform highlighting its amnesic effect.

As far as motor coordination was evaluated, *C. maxima* 100 and 200 mg/kg showed improvement in muscle activity by an increase in the fall time in rotarod. Diazepam 3 mg/kg exhibited a greatly substantial reduction in fall time on the 8th day at both rpm, whereas a highly substantial reduction in fall on the 21st day at both rpm. Imipramine 20 mg/kg showed a greatly substantial increment in fall time on the 8th and 21st days at high speed in comparison with control.

In the SRT, the animals given *C. maxima* seed extract revealed an extremely substantial decline in the time to attain the stage at 24 hours and eighth day, whereas a substantial reduction in the time to achieve the stage on the twenty-first day in comparison with the control. *C. maxima* at 100 mg/kg showed a greatly substantial fall in time to attain the stage at 24 hours, 8th day, and 21st day in comparison with control. *C. maxima* at 200 mg/kg showed a greatly substantial increment in time to attain the stage on the eighth day and a substantial fall in time to attain the stage on the twenty-first day in comparison with the control. Diazepam showed a greatly substantial increase in time to attain the stage highlighting its effect on reduction in motor learning due to muscle relaxant activity.

The antidepressant effects of seeds were determined and evaluated by the forced swimming and tail suspension tests in mice. *C. maxima* extracts showed maximum antidepressant effects at 200 mg/kg comparable with imipramine. This may be due to increased concentration of NA and dopamine and a decrease in the metabolism of NA indicated by decreasing concentration of HVA in the brain, whereas at 200 mg/kg *C. maxima* also revealed an elevation in 5HT level. Dopamine metabolism was also found to increase after *C. maxima* dosing. The reason may be due to CNS active metabolites, such as polyphenols and *β*-carotene [39].

Sinha et al. [40] reported that ethanol extract of *C. maxima* is a cholinesterase inhibitor, hence producing a neuroprotective effect since the hydrolysis of the acetylcholine by cholinesterase has been linked with cognition impairment. Increased activity of the brain AChE causes fast hydrolysis of acetylcholine in turn increases the risk for the progression of dementia. Therefore, there is a growing interest in novel cholinesterase inhibitors for the management of cognition impairment [41]. In addition, higher inhibition of butyrylcholinesterase (BChE) activity is often desirable in humans. Any mutilation of the monoaminergic neurotransmission by monoamine oxidase (MAO) inhibitor has been implicated in the pathogenesis and progression of several neurodegenerative diseases, especially Parkinson's and Alzheimer's [42]. Hence, inhibition of MAO activities by suitable agents, particularly plant-derived molecules/extracts, may provide a useful therapeutic strategy in the management of cognitive impairment. Many previous investigations have reported that plant extracts are potent inhibitors of MAO activity. In this study, the ability of the tested extracts of *C. maxima*, to inhibit MAO activity could be a probable mechanism, contributing to their neuroprotective properties [40]. Hence, inhibition of AChE and BChE activities and stimulation of Na^+^/K^+^-ATPase activity by tested extracts can provide an avenue for the development of effective drugs of plant origin for the management of cognitive disorders.

Arora et al. [43] revealed prominent anxiolytic activity of *Cucurbita moschata* seed extracts at 200 mg/kg which was comparable with the standard drug alprazolam in both models. Moreover, an alteration was also observed in motor coordination by the ethanol extract at the same dose. The probable mechanism suggested was an increase in chloride ion influx suggesting a *γ*-Aminobutyric acid type A (GABAA) receptors-mediated mechanism of action. Hence, it may be assumed that *C. maxima* extract may have produced the effect in a similar pattern.

Antidepressant and anxiolytic drugs have a memory suppressing effect [44]. They emphasize the discovery of newer therapies without adverse effects. Hence in the current study, nootropic effects of *C. maxima* were measured using three different models stationary rod, passive avoidance, and water maze test. *C. maxima* seed extract exhibited an increasing effect on the memory-recalling process both at short-term and long-term levels in comparison with control in all three selected models.

*C. maxima* is popular for the amelioration of various diseases since contain different secondary polyphenolic molecules, such as quercetin and *p*-coumaric acid [40]. Several biological activities of *C. maxima* have been reported due to the presence of these metabolites. It behaves like a potent antioxidant because of a high percentage of *β*-carotene that enhances immunity and decreases the incidence of other medical problems, such as cancer and the progression of heart disease.

Hence in the present study, it is justified to conclude that the reduction in anxiety and depression may be linked with changes in levels of biogenic amines. Recently, a study was conducted on the neuroprotective effect of *C. maxima* ether seed extracts on ethidium bromide-induced demyelination in Wistar rats. The results of this study are concurrent to our study, which shows that *C. maxima* seeds have a potential neuroprotective effect in rat-induced demyelination with an improvement in muscle strength and coordination [45]. Sinha et al. [40] reveal that modulation of MAOs, cholinesterase, and sodium–potassium ATPase activities in the brain through phyto-molecules has been effective in the management of cognitive disorders. Sinha et al. [40] also showed that ethanol and hexane extracts of *C. maxima* at 50 *μ*g/ml concentration inhibited the AChE and BChE activities compared with the standard drug, donepezil which was linked to the presence of quercetin in *Cucurbita* species. Quercetin is a plant-derived polyphenol having anti-carcinogenic, anti-inflammatory, and antiviral properties, as well as the capacity to reduce lipid peroxidation, platelet aggregation, and capillary permeability [46].

## 5. Conclusion

Neurological problems are an increasing trend these days. Therefore, it is a need for time to identify natural products with neuropharmacological benefits. *C. maxima* has now been regarded as an important neuroprotective since several investigators have reported that it contains many neuroprotective and anti-inflammatory metabolites. Thus, it should be included in our daily diet not only to enhance memory, but also to reduce the symptoms of anxiety and depression. Present work authenticates that the chronic use of *C. maxima* seed in neurological problems has been very efficacious in ameliorating such problems. However, before being used widely, adequate clinical trials are crucial. Moreover, without appropriate coverage by reputable worldwide venues, the advantages of these studies cannot be widely distributed.

## Figures and Tables

**Table 1:** Effect of *C. maxima* seed extracts on weight in mice.

Groups/dose (mg/kg)	Weight day 0 (g)	Weight day 8 (g)	Weight day 15 (g)	Weight day 30 (g)	% Change in weight
Control	28 ± 0.26	27 ± 1.15	28 ± 0.47	26 ± 1.08	−7
*C. maxima* 50	26 ± 0.43	25 ± 0.43	28 ± 0.70	26 ± 0.60	0
*C. maxima* 100	28 ± 0.40	26 ± 0.51	29 ± 0.49	25 ± 0.45∗∗	−11
*C. maxima* 200	27 ± 0.78	26 ± 1.05	27 ± 0.92	24 ± 0.78∗∗	−11
Diazepam 3	21 ± 0.62	26 ± 1.02	24 ± 1.43	24 ± 0.32∗∗	10
Imipramine 30	26 ± 0.48	26 ± 1.37	30 ± 2.6	31 ± 2.6∗∗	16

*n* = 10, average ± SEM; ∗∗greatly substantial as compared with initial weight; − (ve) sign indicates a reduction in weight.

**Table 2:** Outcome of *C. maxima* seeds on memory by passive avoidance.

Groups and doses (mg/kg)	Latency time (seconds)			
	3 hours	24 hours	8th day	21st day
Control	115 ± 34	99 ± 11.6	94 ± 15	55 ± 6.26
*C. maxima* 50	253 ± 32∗∗	166 ± 45	286 ± 14∗∗	117 ± 35
*C. maxima* 100	258 ± 28∗∗	282 ± 18.1∗∗	262 ± 26.3∗∗	253 ± 32∗∗
*C. maxima* 200	243 ± 29∗	233 ± 36∗∗	300 ± 0.0∗∗	215 ± 32∗
Diazepam 3	155 ± 65	86 ± 13.5	16 ± 3.1∗	32 ± 14∗
Imipramine 30	257 ± 43∗	245 ± 54.6∗	269 ± 31∗	209 ± 39∗

*n* = 10, average ± SEM; ∗*P* < 0.05 substantial; ∗∗*P* < 0.01 greatly substantial in comparison with control.

**Table 3:** Effect of *C. maxima* seeds on the memory (water maze test).

Groups and doses (mg/kg)	Time to arrive at the stage (seconds)			
	After learning	24 hours	8th day	21st day
Control	14.4 ± 1.5	10.3 ± 0.7	19 ± 0.9	20.3 ± 0.7
*C. maxima* 50	5.0 ± 0.7	2.5 ± 0.6∗∗	4.2 ± 0.5∗∗	4.8 ± 0.8∗
*C. maxima* 100	6.1 ± 0.8	3.9 ± 0.7∗∗	5.5 ± 0.7∗∗	4.9 ± 1∗
*C. maxima* 200	6.0 ± 0.5	3.0 ± 0.6∗∗	3.9 ± 0.5∗∗	4 ± 0.5∗
Diazepam 3	15.2 ± 0.8	33.8 ± 2.7∗∗	36.6 ± 2.9∗∗	91.7 ± 13.1∗∗
Imipramine 30	13.8 ± 1.3	11.9 ± 1.5	10.8 ± 1.7∗∗	6.2 ± 0.9∗∗

*n* = 10, average ± SEM; ∗*P* < 0.05 substantial; ∗∗*P* < 0.01 greatly substantial in comparison with control.

**Table 4:** Effect of *C. maxima* seed extracts on motor coordination by rotarod.

Groups and doses (mg/kg)	Fall time(s) on 10 rpm		Fall time(s) on 30 rpm	
	8th day	21st day	8th day	21st day
Control	135.4 ± 7.2	123.83 ± 16.0	8.77 ± 0.7	4.37 ± 1.06
*C. maxima* 50	154.6 ± 8.8	161.2 ± 7.8	23.9 ± 0.9∗∗	27.4 ± 1.5∗∗
*C. maxima* 100	174.8 ± 2	176 ± 1.5∗	27.1 ± 1.3∗∗	29.7 ± 0.9∗∗
*C. maxima* 200	167.4 ± 7.2	167.3 ± 7.3∗	36.2 ± 1.8∗∗	36.9 ± 1.4∗∗
Diazepam 3	19.2 ± 2.5∗	40.4 ± 5.3∗∗	4.6 ± 0.9∗	4.40 ± 1.08∗∗
Imipramine 30	139 ± 7.2	155.5 ± 1.7	26.3 ± 2.3∗	27.6 ± 3.7∗∗

*n* = 10, average ± SEM; ∗*P* < 0.05 substantial; ∗∗*P* < 0.01 greatly substantial in comparison with control.

**Table 5:** Effect of *C. maxima* seed extract on motor skilled learning and memory (stationary rod).

Groups and doses mg/kg	Time to reach the stage (seconds)			
	After learning	24 hours	8th day	21st day
Control	12.0 ± 1.0	19.5 ± 1.1	25.9 ± 0.8	26.3 ± 1.1
*C. maxima* 50	11.9 ± 0.3	8.8 ± 1.1∗∗	15 ± 1.4∗∗	11 ± 0.9∗
*C. maxima* 100	14.8 ± 0.8	9.2 ± 0.8∗∗	12 ± 0.6∗∗	11 ± 0.9∗∗
*C. maxima* 200	16.2 ± 0.8	13.1 ± 0.8	11.9 ± 0.6∗∗	64.1 ± 9.2∗
Diazepam 3	16.3 ± 0.6	28 ± 5	63.4 ± 8.5∗∗	82.9 ± 10.5∗∗
Imipramine 30	13.9 ± 1.6	11.9 ± 1.8	8.2 ± 1.5∗∗	6.2 ± 0.9∗∗

*n* = 10, average ± SEM; ∗*P* < 0.05 substantial; ∗∗*P* < 0.01 greatly substantial in comparison with control.

**Table 6:** Effect of *C. maxima* seeds on anxiety by light and dark model.

Groups and doses (mg/kg)	No of transitions		% Time in light	
	8th day	21st day	8th day	21st day
Control	1.5 ± 0.2	2.4 ± 0.5	6.7 ± 1.8	10.6 ± 1.7
*C. maxima* 50	6.2 ± 0.9∗∗	7.6 ± 0.7∗∗	30.9 ± 9.6	34.3 ± 3.7∗∗
*C. maxima* 100	4.9 ± 0.3∗∗	4.6 ± 0.4	58.9 ± 7∗∗	55 ± 7∗∗
*C. maxima* 200	3.8 ± 0.3	3.5 ± 0.3	40.3 ± 4.5∗∗	40.5 ± 4.5∗∗
Diazepam 3	4.5 ± 0.5	0.7 ± 0.7	33.5 ± 8.5∗∗	54.3 ± 3.9∗∗
Imipramine 30	5.6 ± 0.9∗	3.8 ± 1.1	10.8 ± 2.7	6.3 ± 2

*n* = 10, average ± SEM; ∗*P* < 0.05 substantial; ∗∗*P* < 0.01 greatly substantial in comparison with control.

**Table 7:** Effect of *C. maxima* seed extracts on anxiety (elevated plus maze).

Groups and doses (mg/kg)	Open arm's time (seconds)		Closed arm's time (seconds)		Closed-arm entries		Open arm entries		Anxiety index	
	8th day	21st day	8th day	21st day	8th day	21st day	8th day	21st day	8th day	21st day
Control	11 ± 1.28	27 ± 3.82	289 ± 1.3	270.8 ± 4.5	2.9 ± 0.5	3.6 ± 0.4	3.3 ± 0.6	3.8 ± 0.6	0.71	0.68
*C. maxima* 50	70 ± 8	23 ± 3	230 ± 8	277 ± 3	4.4 ± 0.8	2.5 ± 0.4	7.4 ± 1.3	4.3 ± 0.7	0.58	0.65
*C. maxima* 100	187 ± 28.5∗∗	91.6 ± 14.5∗	113 ± 8.5∗∗	209 ± 14.5∗	3.8 ± 0.6	4 ± 0.71	8.5 ± 1.4∗∗	8.0 ± 1.3	0.33	0.51
*C. maxima* 200	214 ± 22∗∗	93 ± 8.5∗	86 ± 22∗∗	206.9 ± 8.5∗	2.7 ± 0.6	4.0 ± 0.7	8.4 ± 1.3∗∗	8.4 ± 1.7	0.26	0.49
Diazepam 3	181 ± 24∗∗	192 ± 35∗∗	119 ± 24∗∗	107.6 ± 35∗∗	2.8 ± 0.5	6.3 ± 0.5∗	5.3 ± 0.5	10.0 ± 1.2∗	0.37	0.39
Imipramine 30	26 ± 6.5	47 ± 15	266.2 ± 12	253 ± 15	4.5 ± 1.3	4.0 ± 1.9	7.8 ± 2	8.0 ± 1.6	0.63	0.59

*n* = 10, average ± SEM; ∗*P* < 0.05 substantial; ∗∗*P* < 0.01 greatly substantial in comparison with control.

**Table 8:** Effect of *C. maxima* seed extracts on head dips.

Groups and doses (mg/kg)	No. of head dips	
	8th day	21st day
Control	31.5 ± 0.9	29.7 ± 0.6
*C. maxima* 50	28 ± 1.5	28.5 ± 1.3
*C. maxima* 100	26.3 ± 1.5	15.5 ± 2.2∗∗
*C. maxima* 200	24 ± 1.4∗	17 ± 1.9∗∗
Diazepam 3	39.8 ± 1.7∗	21 ± 1.8∗∗
Imipramine 30	19.4 ± 2.9∗∗	17.4 ± 2.0∗∗

*n* = 10, average ± SEM; ∗*P* < 0.05 substantial; ∗∗*P* < 0.01 greatly substantial in comparison with control.

**Table 9:** Outcome of *C. maxima* seeds in open field test.

Groups and doses (mg/kg)	Total distance travelled (cm)		No. of center entries		Centre time(s)		No. of rearing		Duration of rearing (s)	
	8th day	21st day	8th day	21st day	8th day	21st day	8th day	21st day	8th day	21st day
Control	1904 ± 81	2273 ± 14	2.8 ± 0.3	2.7 ± 0.3	3.4 ± 0.5	3.5 ± 0.6	4.3 ± 0.5	11 ± 1	4.5 ± 0.5	13 ± 1
*C. maxima* 50	3007 ± 11∗∗	2140 ± 15	5.8 ± 0.7∗	8 ± 0.9∗∗	5 ± 1	8 ± 1.2s	4.7 ± 1	12 ± 1.5	17 ± 1.2∗∗	24 ± 1.2∗
*C. maxima* 100	2642 ± 14∗	2412 ± 16	8 ± 0.6∗∗	8.6 ± 0.6∗∗	14 ± 1∗∗	14 ± 1.1∗∗	16.3 ± 0.8∗∗	25 ± 1∗∗	16 ± 1∗	32 ± 1.8∗∗
*C. maxima* 200	2941 ± 11∗∗	2572 ± 11	7.8 ± 0.3∗∗	11 ± 0.7∗∗	15 ± 1∗∗	17 ± 1.6∗∗	26.5 ± 0.9∗∗	24 ± 1∗∗	12.8 ± 1∗∗	32 ± 4.2∗∗
Diazepam 3	2452 ± 22	1246 ± 18∗	6.25 ± 1	10 ± 1∗∗	6 ± 2	23 ± 2∗∗	2 ± 0.5	1 ± 0.3∗∗	3 ± 0.9	5.5 ± 1∗
Imipramine 30	1503 ± 80	1591 ± 83	2 ± 0.28	6.5 ± 0.6∗∗	3 ± 0.6	7.32 ± 0.4	15 ± 1∗∗	22 ± 0.7∗∗	8 ± 1.9	11 ± 2

*n* = 10, average ± SEM; ∗*P* < 0.05 substantial; ∗∗*P* < 0.01 greatly substantial in comparison with control.

**Table 10:** Effect of *C. maxima* seed extracts on depression by forced swim test and tail suspension test.

Groups and doses (mg/kg)	Forced swim test		Tail suspension test	
	Immobility time (seconds) 22nd day	% Reduction immobility	Immobility time (seconds) 22nd day	% Reduction immobility
Control	200 ± 2.3	—	211.2 ± 4.8	—
*C. maxima* 50	154 ± 7.8∗∗	36	158 ± 1.7∗∗	34.3
*C. maxima* 100	155 ± 8.7∗∗	35.4	155 ± 2.5∗∗	35.4
*C. maxima* 200	122 ± 7.8∗∗	49.3	139 ± 4.1∗∗	42
Diazepam 3	218 ± 14.8	9.19	224 ± 4.1	2.5
Imipramine 30	110 ± 6.3∗∗	54.1	113.9 ± 8.6∗∗	52.5

*n* = 10, average ± SEM; ∗*P* < 0.05 substantial; ∗∗*P* < 0.01 greatly substantial in comparison with control.

**Table 11:** Outcome of *C. maxima* seed extracts on brain biogenic amines.

Groups and doses (mg/kg)	NA	HVA	DA	DOPAC	5HT	5-HIAA
	(pg/mg of wet tissue)					
Control	56 ± 4.4	487 ± 9.5∗∗	357 ± 128.5	172 ± 29.8	97 ± 29.5	333 ± 7.8
*C. maxima* 50	347 ± 10.5∗∗	298.8 ± 18.5∗∗	435.7 ± 17.2	130.2 ± 12.5	313 ± 29.2	325 ± 53.3
*C. maxima* 100	573 ± 21∗∗	264.5 ± 41∗∗	632 ± 13.5∗∗	421 ± 13∗∗	382 ± 83.2	269.2 ± 75.2
*C. maxima* 200	697.8 ± 35∗∗	423.2 ± 74	883.1 ± 58∗∗	443.9 ± 52∗∗	452.6 ± 86∗∗	296.0 ± 34

*n* = 10, mean ± SEM; ∗*P* < 0.05 substantial; ∗∗*P* < 0.01 greatly substantial in comparison with control; NA = norepinephrine; DOPAC = dioxyphenylacetic acid; DA = dopamine; 5-HIAA = 5-hydroxyindoleaceticacid; HVA = homovanillic acid; 5HT = 5-hydroxytryptamine.

## Data Availability

Data supporting this research article are available from the corresponding author or first author on reasonable request.
